# Selective Interventricular Septal Radiofrequency Ablation in Patients With Hypertrophic Obstructive Cardiomyopathy: Who Can Benefit?

**DOI:** 10.3389/fcvm.2021.743044

**Published:** 2021-11-16

**Authors:** Qiang Liu, Hangyuan Qiu, Ruhong Jiang, Xiaomei Tang, Wenpu Guo, Kuangshi Zhou, Qiufan Chen, Yaxun Sun, Lu Yu, Shiquan Chen, Pei Zhang, Xia Sheng, Jun Zhu, Jianwei Lin, Hui Cheng, Yunhe Wang, Bei Wang, Chan Yu, Yankai Mao, Juhong Zhang, Zuwen Zhang, Paul C. Zei, Guosheng Fu, Chenyang Jiang

**Affiliations:** ^1^Department of Cardiology, Sir Run Run Shaw Hospital, School of Medicine, Zhejiang University, Hangzhou, China; ^2^Medicine, Brigham and Women's Hospital Associate, Harvard Medical School, Boston, MA, United States

**Keywords:** hypertrophic obstructive cardiomyopathy, radiofrequency septal ablation, anterior mitral leaflet, papillary muscle displacement, mid-septal hypertrophy

## Abstract

**Introduction:** Septal mass reduction is beneficial for hypertrophic obstructive cardiomyopathy (HOCM) patients with severe left ventricular outflow (LVOT) gradient and symptoms, with surgical myectomy or alcohol septal ablation (ASA) currently recommended in selected patients. Radiofrequency (RF) ablation of hypertrophied septum has been published as a novel method to alleviate LVOT obstruction in small populations. This study aims to investigate factors influencing clinical outcomes of radiofrequency septum ablation.

**Methods and Results:** In this study, 20 patients with HOCM who underwent endocardial ablation were included. Echocardiography and cardiac MRI (CMR) data was collected and analyzed pre- and (or) post- procedure. Nineteen patients underwent ablation successfully, while ablation was aborted in one patient with prior RBBB due to transient complete atrioventricular block (AVB). After 6 months of follow-up, NYHA heart functional class improved from III (2 - 3) to II (1 - 2) (*p* < 0.001), and resting LVOT gradient was significantly reduced (87.6 ± 29.5 mmHg vs. 48.1 ± 29.7, *p* < 0.001). LVOT gradient reduction was significantly higher in patients with limited basal septal hypertrophy (60.9 ± 8.3 vs. 27.9 ± 7.1, *p* = 0.01), shorter anterior mitral leaflet (56.1 ± 6.4 vs. 20.4 ± 5.0, *p* < 0.01), and normally positioned papillary muscle (36.9 ± 7.1 vs. 75.0 ± 6.3, *p* < 0.05).

**Conclusions:** Endocardial septal ablation appears to be a safe and effective procedure for alleviating LVOT gradient in patients with HOCM, especially in those with limited basal septal hypertrophy, shorter anterior mitral leaflet, and normal positioned papillary muscle.

## Introduction

Hypertrophic cardiomyopathy (HCM) is a heterogeneous monogenic heart disease characterized by a small left ventricular cavity and marked hypertrophy of the myocardium, with a prevalence of 0.2–0.5% around the world. Obstruction of the left ventricular outflow tract (LVOT) is a major hallmark of HCM, present in ~2-3rds of patients, classified as hypertrophic obstructive cardiomyopathy (HOCM). Reduction of LVOT gradients has been shown to improve symptoms and possibly prognosis (1–3). For patients with severe and highly symptomatic LVOT obstruction despite medication, transaortic surgical myomectomy has been considered the gold standard for many years. Alcohol septal ablation (ASA) has been utilized recently as a minimally invasive alternative to surgical myectomy (4–6). Experience with permanent pacemaker (PPM) implantation to force RV pacing and RV/LV dys-synchrony as another treatment has fallen out of favor (7, 8).

Recently, endocardial catheter-based septal radiofrequency ablation of the LVOT has been utilized to treat HOCM. Catheter ablation, particularly with utilization of an electroanatomic mapping system and/or intracardiac echocardiography (ICE), may allow a higher degree of ablative accuracy, resulting in potentially more effective relief of obstruction and less risk of collateral myocardial and other injury. Single-center evidence has demonstrated that endocardial radiofrequency ablation for septal hypertrophy might be an option for patients with HOCM in order to alleviate the LVOT gradient (9–14). The accuracy of tissue damage and improvement in LVOT gradient, symptoms, and quality of life in this preliminary group of patients appears promising (9). However, to date, the selection of appropriate patients for this procedure remains unclear. In this study, we collected data from patients who underwent septal ablation and assessed the factors that may influence treatment outcomes.

## Materials and Methods

### Patients Selection

Twenty consecutive patients with HOCM and LVOT gradients of ≥50 mmHg at rest and drug-refractory symptoms (NYHA class II or III) were sequentially enrolled. The protocol was approved by the ethics committee of Sir Run Run Shaw Hospital. All patients provided full informed consent. All echocardiography and CMR data was anonymized, and no identifiable patient data were used.

### Imaging Study

LVOT gradient was measured *via* transthoracic echocardiograph under resting conditions before the procedure and at 1, 3, and 6 months post procedure during follow-up. Other variables measured included maximal interventricular septum (IVS) thickness, left atrial (LA) diameter, and anterior mitral valve leaflet (AML) length. LVOT obstruction due to systolic anterior motion of the mitral valve (SAM) was determined by the highest gradient acquired with continues wave Doppler in the apical three-chamber (or five-chamber) view. The length of the AML was measured in diastole on the apical three-chamber view (15). Considering the severity of symptoms and significant gradient, stress echocardiography was not performed.

**Figure 1:**
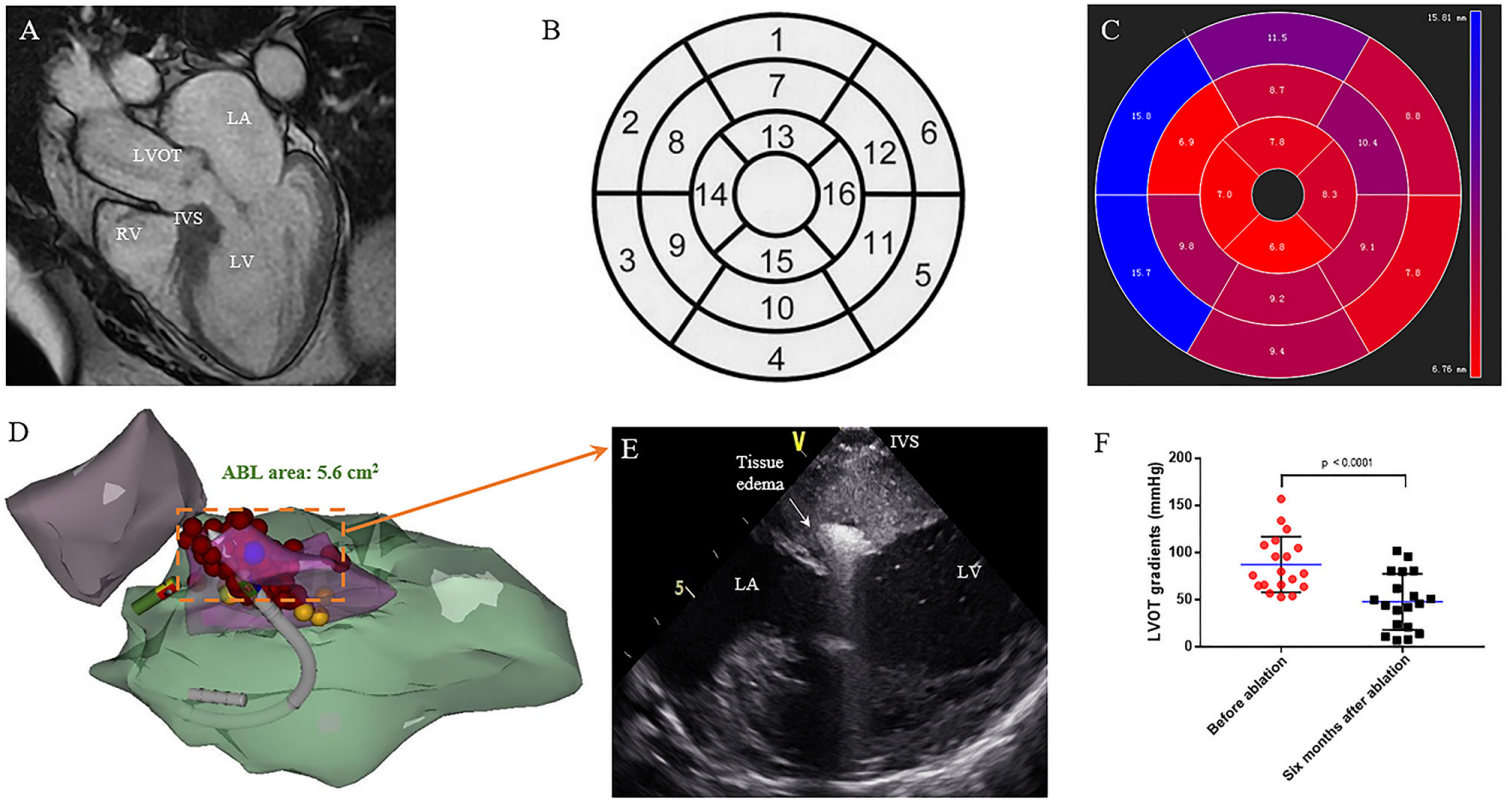
Interventricular septal ablation in patients with HOCM. **(A)** CMR end-diastolic long-axis image demonstrates a significant hypertrophy in basal septum. **(B)** Display of the 16 myocardial segments on a circumferential polar plot. The recommended nomenclature for tomographic imaging of the heart: 1. Basal anterior, 2. Basal anteroseptal, 3. Basal inferoseptal, 4. Basal inferior, 5. Basal inferolateral, 6. Basal anterolateral, 7. Mid anterior, 8. Mid anteroseptal, 9. Mid inferoseptal, 10. Mid inferior, 11. Mid inferolateral, 12. Mid anterolateral, 13. Apical anterior, 14. Apical septal, 15. Apical inferior, 16. Apical lateral. **(C)** The circumferential polar plot presents a limited hypertrophy in basal anteroseptal and inferoseptal segments. The number indicates the average thickness of each segment. **(D)** Right anterior oblique (RAO) projection of the CARTO shell. Left ventricular (green), aorta (purple), SAM area of hypertrophic septum (pink), and left bundle branch (yellow) are presented. Ablation is performed on SAM area (red). **(E)** On the live ICE screen, significant tissue edema can be observed after ablation. **(F)** Reduction of obstruction at rest is seen on continuous-wave Doppler echocardiography. The LVOT gradient (in mmHg) is significantly decreased after 6 months follow up. (*P* = 0.001) LA, left atrium; LV, left ventricle; LVOT, left ventricular outflow tract; RV, right ventricle; IVS, interventricular septum.

Considering the ablation procedure will not lead to significant changes in LV morphology (9), CMR was performed either before or after the procedure with patient's permission. Distribution of hypertrophic segments was assessed using 16-segment model (16). Average septal thickness measurements in each of the 16 segments were automatically calculated with commercially available software (Figure 1). At the 3 LV levels (i.e., basal, mid, and apical), the number of hypertrophied segments in septum (≥15 mm) was recorded as well as the papillary muscle (PM) abnormalities (the PM was defined as anterior positioned when more than half of it originated from the anterior wall above the line bisecting the LV cavity into two equal halves) (15).

### Radiofrequency Ablation

All procedures were performed under conscious sedation using fentanyl and midazolam. Invasive arterial blood pressure was monitored. The SoundStarTM catheter (BiosenseWebster, Diamond Bar, CA, USA) was inserted *via* the left femoral vein and manipulated into the right ventricle (RV). The phased array probe was used to determine the ICE geometries of the RV, LV, and aorta. Endocardial borders and aortic cusps were delineated and transferred into the CARTO system (BiosenseWebster). The hypertrophic septum was intentionally constructed in detail during systole (Figure 1). A quadripolar catheter was used to indicate the location of the His bundle and for back-up RV pacing. A retrograde aortic approach to the LV was preferred, while trans-atrial septal access was used if the retrograde aortic approach could not reach stable contact. Intravenous heparin (100 unit per kg) was administered to keep the activated clotting time 300–350 s. The locations of the His bundle and left bundle were annotated on the CARTO shell (Figure 1). RF energy was delivered *via* a Navistar THERMOCOOL catheter or Smartouch catheter (Biosense Webster). Using a combination of CARTO and intracardiac echo navigation, RF energy was delivered to the SAM-septal contact area (Figure 1), with power of 35–50 W, limited temperature of 43°C, and irrigation rate at 30 mL/min. If there was no elevation of arterial BP after 30 s ablation, the charge would be terminated. If a significant arterial BP elevation could be observed, RF ablation would be extended to 60–120 s until no further elevation of arterial BP. Every effort was made to avoid injury to the His bundle/left bundle/fascicles during ablation. The invasive resting LVOT gradients were monitored during the procedure. The procedural endpoint was an LVOT gradient reduction of >50% or complete ablation of the SAM-septal contact area (9). Methylprednisolone was administered for the next 3 days after the procedure to alleviate edema.

### Follow-Up

After the procedure, anticoagulant drugs including DOAC or warfarin were used for 3 months. Echocardiography and surface ECGs were performed immediately after the procedure and then at 1, 3, and 6 months after discharge. Furthermore, ECG and 24-h Holter recordings were performed anytime patients experienced symptoms suggestive of arrhythmia. The 36F quality-of-life index was measured in all patients before and 6 months after the procedure.

### Statistical Analysis

Continuous variables are expressed as the mean ± SD and categorical variables were reported as counts and frequencies. Intergroup differences were compared by using analysis of variance or the Kruskal-Wallis test for overall comparisons and the *T*-test or Mann-Whitney *U*-test for two-group comparisons. Statistical analyses were performed with SPSS statistical software version 11.0 (Cary, North Carolina). All statistical tests were 2 sided, and a *p*-value < 0.05 was considered significant.

## Results

### Baseline Characteristics of the Patients

The baseline characteristics are shown in Table 1. Mean age of the patients was 57.7 ± 14.4 years, with a range between 28 and 82 years, and 30% were men. All patients had a maximal LVOT gradient of >50 mmHg, with severe symptoms. Leading clinical symptoms were dyspnea (New York Heart Association heart functional class III or IV) in 95%, angina (Canadian Cardiovascular Society class III) in 15%, and syncope /presyncope in 10% of the patients. Patient No. 20 had a history of ASA and presented with a right branch bundle block (RBBB) according to ECG. No patient had a history of pacemaker or ICD implantation.

**Table 1:** Baseline characteristics (*n* = 20).

**Age, y**	**57.7 ± 14.4**
**Male, %**	**6 (30)**
**BMI, Kg/m^2^**	**26.1 ± 4.3**
**Dyspnea**	
**NYHA functional class III/IV, %**	**19 (95)**
**Angina pectoris, %**	**3 (15)**
**Syncope or presyncope, %**	**2 (10)**
**Palpitations, %**	**20 (100)**
**Prior septal reduction, %**	
**ASA, %**	**1 (5)**
**Cardiovascular diseases**	
**Hypertension, %**	**10 (50)**
**Coronary artery disease, %**	**2 (10)**
**Atrial fibrillation, %**	**3 (15)**
**Echocardiography**	
**Left atrial diameter, mm**	**42.7 ± 5.7**
**Length of AML**	**27.8 ± 3.4**
**Doppler gradient at rest, mm Hg**	**86.5 ± 29.2**
**Gradient at rest >50 mm Hg, %**	**20 (100)**
**LVEF, %**	**72.5 ± 4.9**
**ECG, including Holter**	
**Sinus rhythm, %**	**18 (90)**
**Right bundle branch block**	**1 (5)**
**Atrial fibrillation, %**	**4 (20)**
**Non-sustained ventricular tachycardia, %**	**2 (10)**

*Values are mean ± SD or n (%).*

*NYHA, New York Heart Association; ASA, alcohol septal ablation; AML, anterior mitral leaflet; LVEF, left ventricular eject; ECG, electrocardiography*.

**Figure 2:**
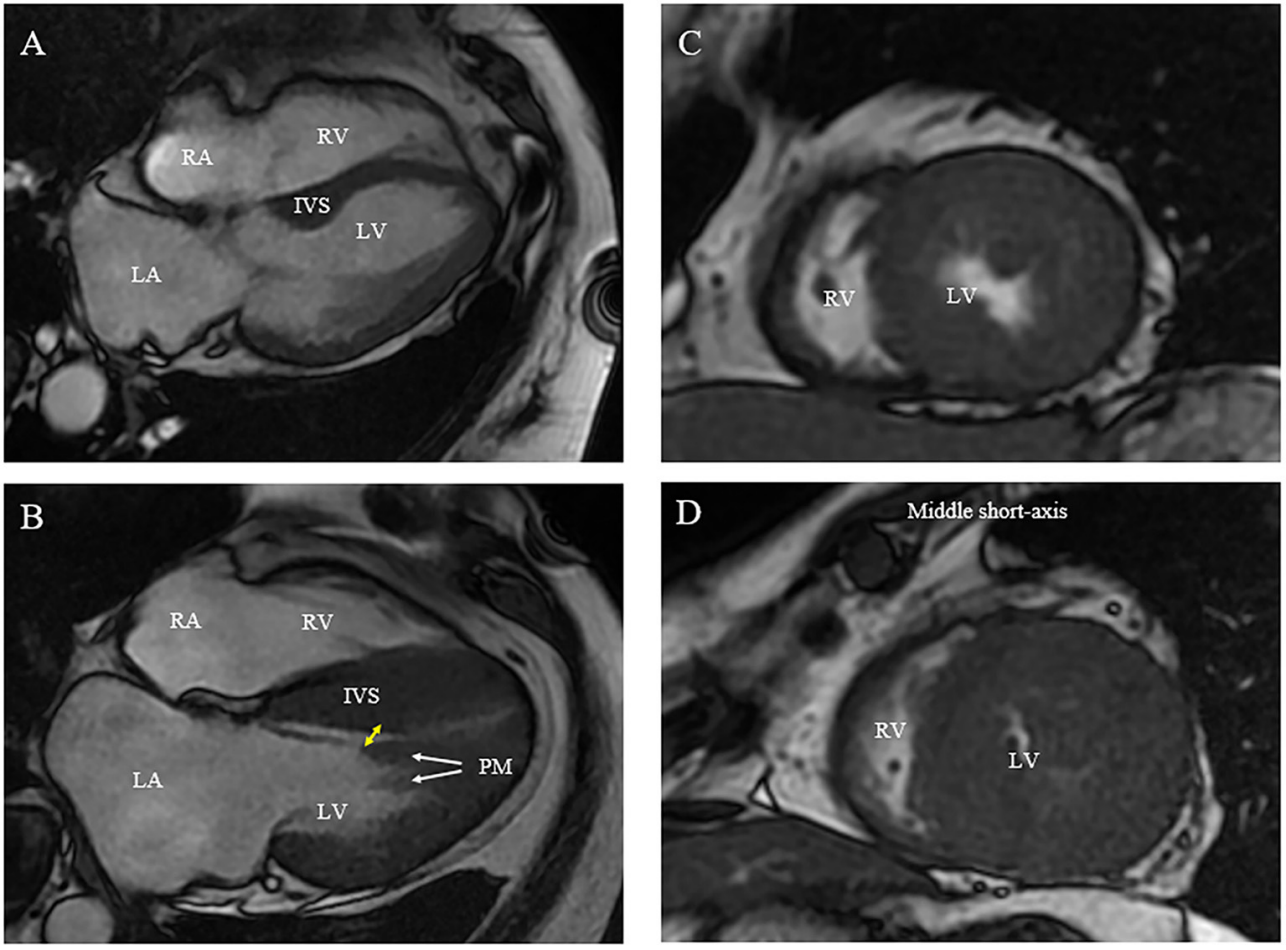
Morphologic abnormalities of papillary muscle (PM) contributing to outflow tract obstruction [**(A)** normal; **(B)** abnormal]. **(A,B)** On CMR, hypertrophied and apically displaced papillary muscle (white arrows) with superior head (yellow arrow) in close proximity to the bulged septum, positioning mitral valve plane toward ventricular septum compared with normal positioned PM **(B)**. **(C,D)** As compared with a relatively normal papillary muscle object **(C)**, CMR end-systolic image presents a severely narrowed LV cavity in patient with hypertrophied papillary muscle **(D)**.

**Figure 3:**
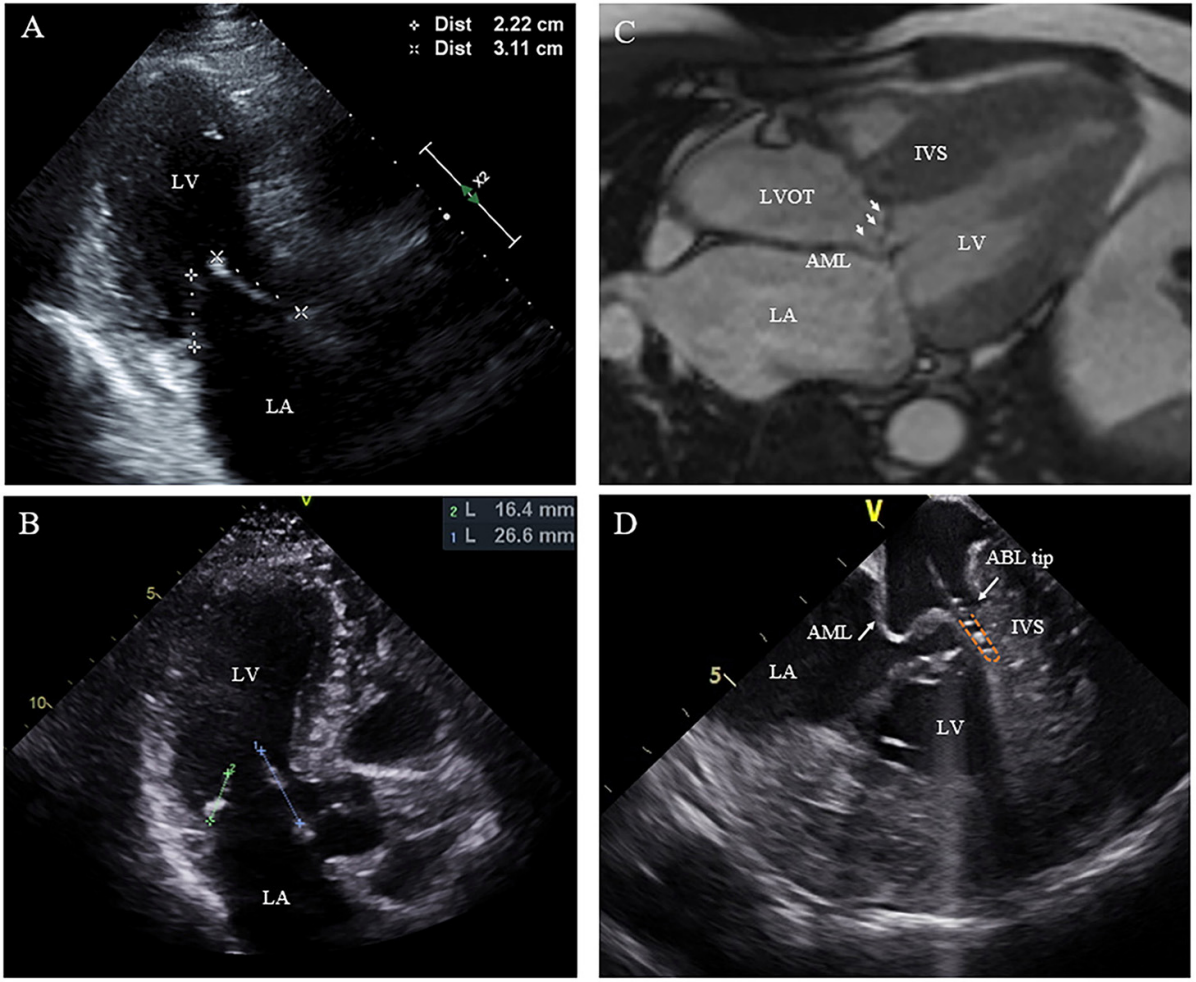
Morphologic abnormalities of mitral valve contributing to outflow tract obstruction. **(A,B)** The length of the AML was measured by echocardiography in diastole on the apical three-chamber view. **(C)** Extraordinarily long anterior mitral valve leaflet on CMR. **(D)** On live ICE screen, the AML (white arrow) is reflexed and attached to IVS during systole and pats on the ablation catheter tip during procedure. AML, anterior mitral leaflet.

**Figure 4:**
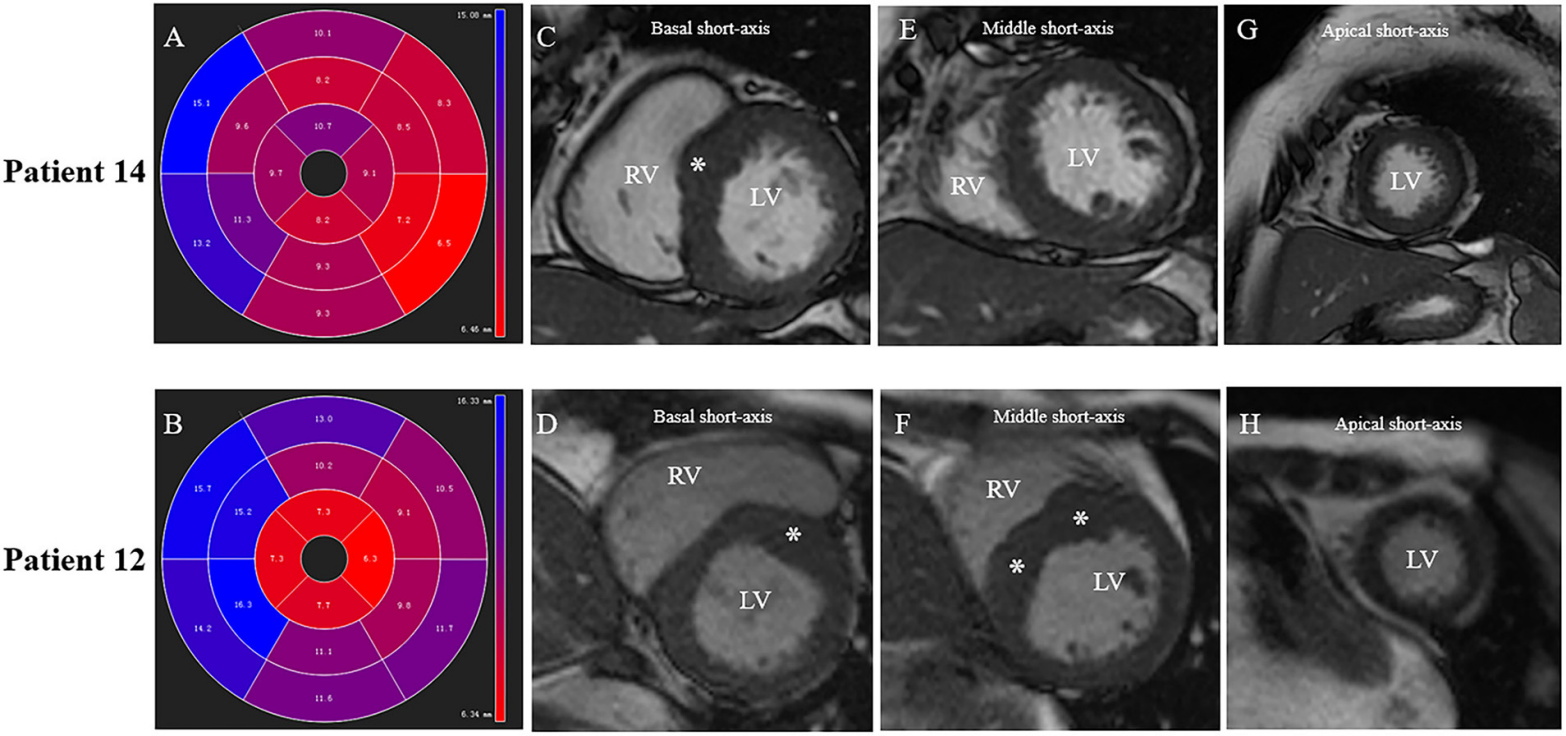
Different patterns of septum hypertrophy among patients. Patient No.12: **(B,D,F,H)**. Patient No.14: **(A,C,E,G)**. **(A,B)** CMR end-diastolic images demonstrating different patterns of interventricular septum hypertrophy. The distribution of LV hypertrophy is limited (basal anteroseptal segment) in **(A)**, while diffused (basal, mid anteroseptal, and mid inferoseptal segments) in **(B)**. **(C,D)** Short-axis view presents a segmental LV hypertrophy of the basal anterior septum in both patients. **(E,F)** Both mid anteroseptal and inferoseptal segments are free of hypertrophy in **(E)**, but present hypertrophied phenotype in **(F)**. **(G,H)** Apical septal segment is free of hypertrophy in both patients.

The echocardiographic and CMR measurements of each patient are shown in Table 2 and Figures 2–4. Maximal septal thickness was 20.1 ± 6.4 mm, left atrial diameter was 42.7 ± 5.7 mm, the AML length was 27.8 ± 3.4 mm and LVOT gradient was 86.5 ± 29.2 mm Hg at rest. In 16 patients with CMR, all had basal IVS hypertrophy and nine of them had mid- or apical IVS hypertrophy (Table 2 and Figure 2). Hypertrophied and anterior displacement of the papillary muscle (DPM) was observed in 12 patients (Table 2 and Figure 2B). No patient had abnormal MV chordal attachments. Patient No. 20 presented with an abnormal morphology of the IVS according to the CMR images. The middle portion of the IVS was flimsy compared with the basal and apical portions due to previous ASA.

**Table 2:** Echocardiography and CMR data in 19 patients.

**No**.	**Echocardiography**						**CMR**	
	**Maximal septal thickness, mm**	**Length of AML, mm**	**LVOT gradient at rest, mm Hg**			**Reduction of gradient, %**	**The distribution of hypertrophied segments in septum**	**Hypertrophied and displacement PM**
			**Before ablation**	**3 days after ablation**	**6 months after ablation**			
1	21.7	28.9	65	160	24	63	2, 3, 9^a^	No
2	15.5	28.2	113	66	81	28	2, 3^a^	Yes
3	15.9	22.0	80	21	7	91	0^a^	No
4	19	20.4	96	87	21	78	-	-
5	19.2	28.2	157	95	102	35	-	-
6	16.4	31.1	72	129	54	25	2, 3, 9^a^	Yes
7	17.2	32.6	64	65	62	3	2, 3, 9	Yes
8	21.5	28.6	134	90	44	67	3	No
9	17.4	28.9	108	87	81	25	2, 3^a^	Yes
10	18.1	28.4	96	11	51	47	2^a^	Yes
11	22.5	26.2	78	62	42	46	2, 3	Yes
12	23.3	32.3	105	117	80	25	2, 8, 9	Yes
13	15.7	28.4	125	85	96	23	2, 3, 9	Yes
14	18.8	26.6	54	11	11	80	2	Yes
15	19.5	26.0	53	20	8	85	0	Yes
16	14.3	28.5	66	50	14	79	0	No
17	41.3	27.6	76	88	46	39	2, 3, 8, 9, 14	Yes
18	15.1	30.2	66	88	50	17	2, 3, 9	Yes
19	32.1	30.4	57	60	39	32	-	-

*The distribution of hypertrophied segments in septum and hypertrophied and displacement PM was measured in CMR. The distribution of hypertrophied segments in septum is recorded according to the nomenclature for tomographic imaging of the heart (Figure 1B).*

*AML, anterior mitral leaflet; PM, papillary muscle; LVOT, left ventricular outflow tract.*

a *CMR is applied after ablation*.

### Mapping and Ablation

Nineteen of the 20 patients underwent ablation successfully, while one patient with RBBB presented with transient complete AVB at the beginning of RF ablation. The procedure was discontinued without further ablation. SAM can be observed in all patients on ICE with the AML either touching or nearly touching the interventricular septum. For patient No. 12, an extraordinarily long and flexible mitral leaflet was observed with real-time ICE. The AML was reflexed and attached to the IVS during systole (Figure 3D). RF energy was delivered only in the same region where the AML flapped. Trans-atrial septal access was used in five patients to achieve more stable catheter-tissue contact. After ablation, significant tissue edema can be observed on the real-time ICE image (Figure 1E). Invasive resting LVOT gradients were measured before and after the procedure and decreased significantly from 88.9 ± 30.5 to 33.5 ± 30.1 mmHg (*p* < 0.001). The mean procedure time was 208.4 ± 49.4 min and the mean X-ray exposure time was 13.9 ± 7.2 min. A mean of 15.2 ± 8.1 min of RF energy was applied. A mean depth of 4.3 ± 1.2 mm tissue damage was created. Ablated area was 2.9 ± 1.4 cm^2^, representing 12.7 ± 5.6% of total septum endocardial surface (Figure 1D).

### Six-Month Follow-Up After Ablation

**Figure 5:**
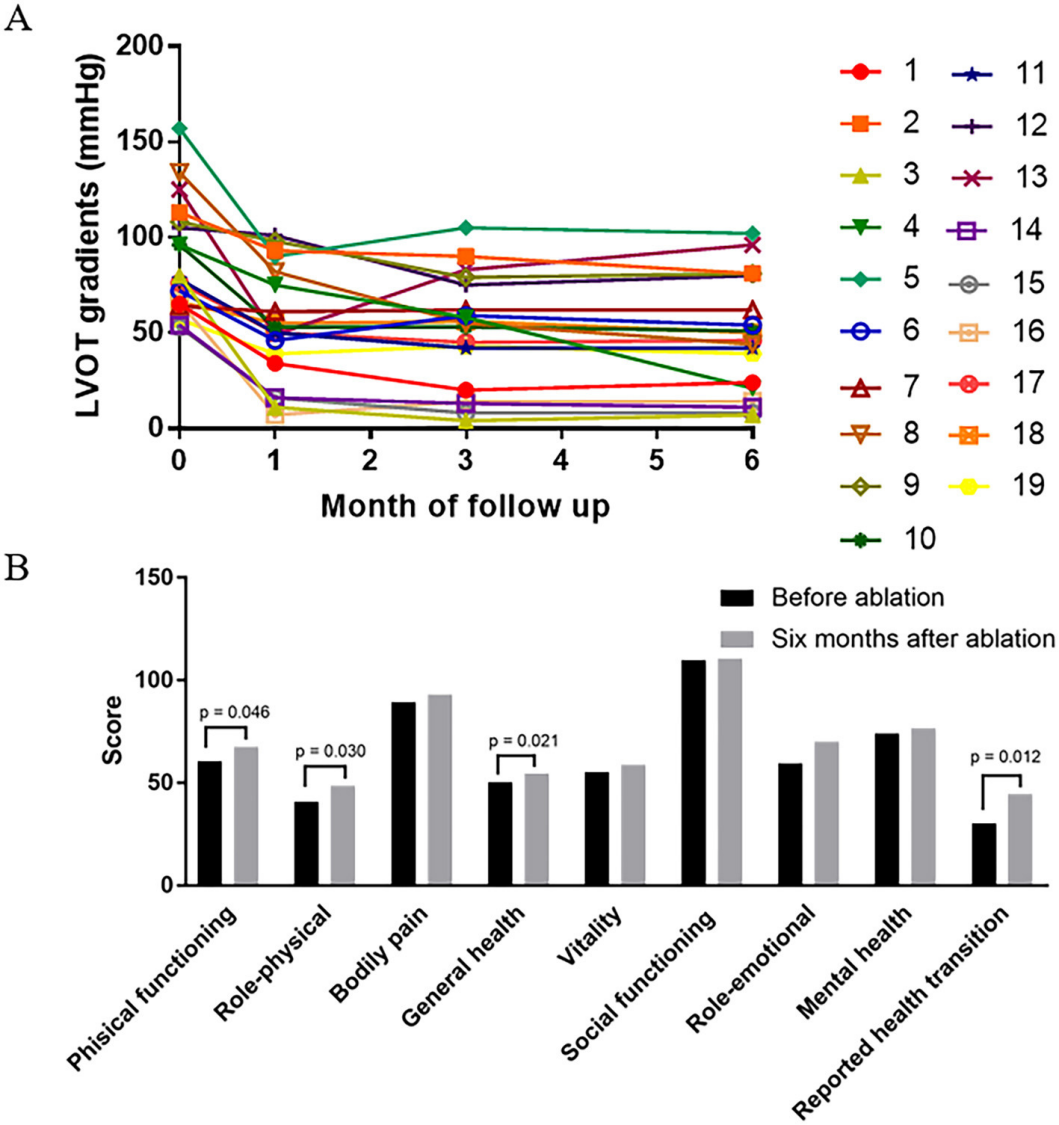
The change of hemodynamic and health-related quality of life after 6 months follow up. **(A)** LVOT gradient is shown (in mmHg) before, 1 month after, 3 months after, and 6 months after LV septal radiofrequency ablation. Reduction of gradient is significant after 6 months follow up (*p* < 0.001) especially in patient 3, 4, 8, 14, 15, and 16. **(B)** Comparison of health-related quality of life before and 6 months after ablation procedure. The score of four dimensions (physical functioning, role-physical, general health, and reported health transition) are increased after 6 months follow up.

All 19 patients demonstrated improvement in symptoms. The NYHA functional class improved from NYHA III (2-3) to NYHA II (1-2) (*p* < 0.001). There was a favorable hemodynamic effect, with reduction or elimination of the outflow obstruction at the 6-month follow-up with the resting LVOT gradient decreased from 87.6 ± 29.5 to 48.1 ± 29.7 mmHg (*p* < 0.001) (Figures 1F, 5A). No significant change was present in the IVS thickness and LA diameter after 6 months of follow-up, respectively (20.3 ± 6.5 vs. 20.1 ± 6.7 mm, *p* = 0.6), (42.6 ± 5.8 vs. 41.7 ± 6.6 mm, *p* = 0.1). Four dimensions (physical functioning, role-physical, general health, and reported health transition) of the SF36 quality-of-life index had values that increased after 6 months follow-up (Figure 5B).

**Figure 6:**
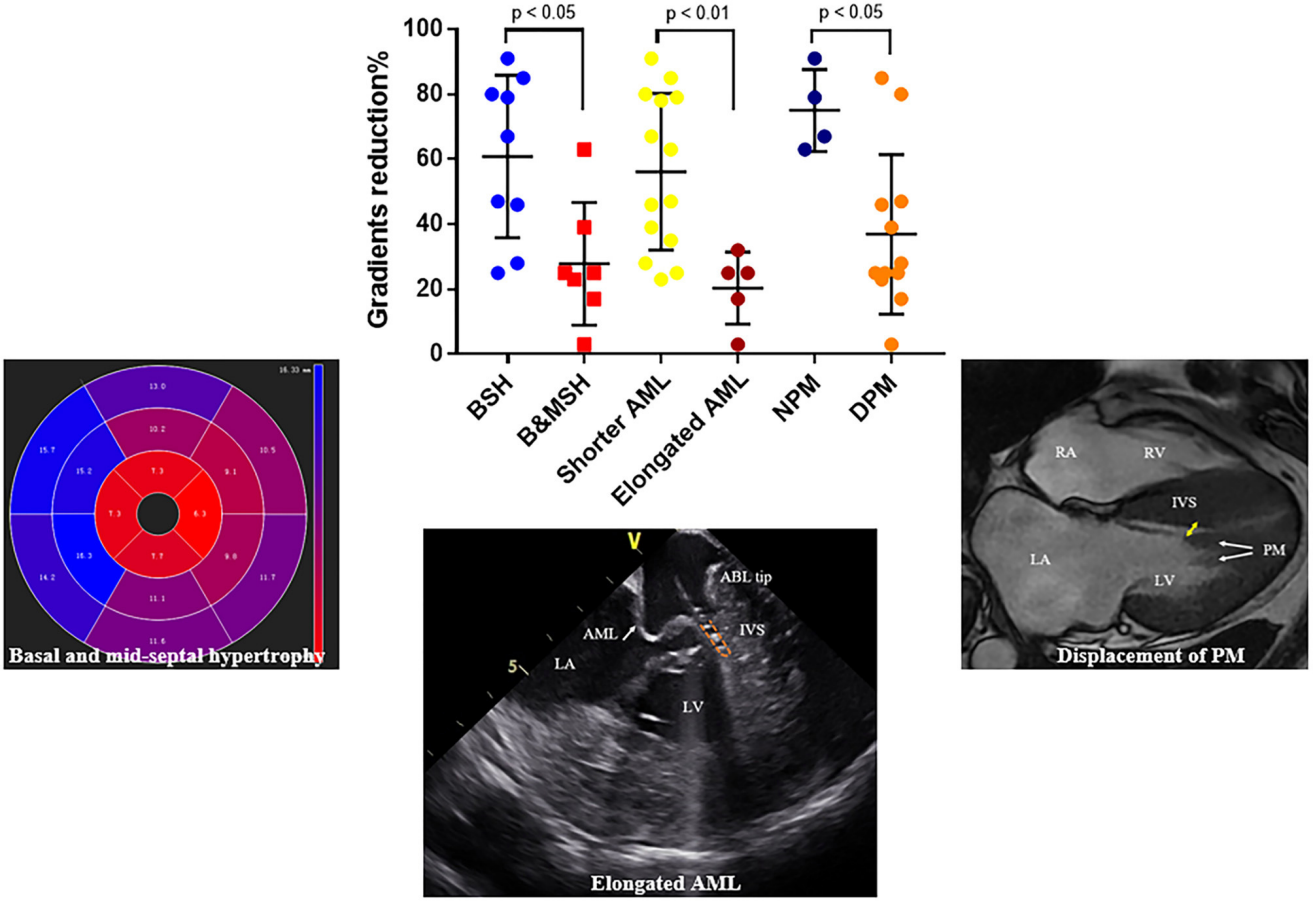
Representative figure. Factors that influence the outcomes of endocardial radiofrequency septal ablation. Reduction of LVOT gradient is significantly higher in patients with limited basal septal hypertrophy, shorter AML and normally positioned PM, respectively. BSH, basal septal hypertrophy; B&MSH, basal and mid-septal hypertrophy; NPM, normally positioned papillary muscle; DPM, anterior displacement of papillary muscle.

The reduction of gradient was greater in patients with limited basal septal hypertrophy (the septal hypertrophy is limited in basal segments; 60.9 ± 8.3 vs. 27.9 ± 7.1 mm, *p* = 0.01) and shorter AML (56.1 ± 6.4 vs. 20.4 ± 5.0 mm, *p* < 0.01). For patients with DPM, the reduction of gradient was lower than others (36.9 ± 7.1 vs. 75.0 ± 6.3mm, *p* < 0.05) (Figure 6).

### ECG Data During Follow-Up

In all 20 patients, no serious complications occurred. Four patients developed a bundle-branch block pattern on surface ECG (2 LBBB, 2 RBBB) within the first 3 days after the procedure. Two recovered at the 6-month follow-up (1 LBBB and 1 RBBB). The duration of the QRS increased significantly from 97.6 ± 16.5 to 109.3 ± 17.4 ms (before ablation vs. 6 months follow up, *p* = 0.003).

## Discussion

For patients with drug-resistant, symptomatic HOCM, mechanical relief in the form of septal reduction therapy is effective to alleviate symptoms and potentially improve prognosis (3). Surgical myectomy remains the gold standard when performed in high-volume centers with good procedural success and low mortality. However, surgical myectomy is morbid and is best performed by experienced operators. For patients with co-morbidities, many centers will choose ASA as first line septal reduction therapy (4). But due to the constraints of septal arterial anatomy, the alcohol-induced infarction may be insufficient and inaccurate, or even too large, resulting in significant and potentially morbid myocardial infarction. In series of multiple studies, up to a third of patients have an unsatisfactory outcome (17, 18). In addition, patients with the unfavorable anatomy of the left ventricle such as a thin septum and concomitant mitral valve abnormalities would be unsuitable for ASA (19).

Endocardial septal ablation by RF has been recently developed to alleviate LVOT obstruction in some centers. After 6 months to 1 year follow up, preliminary results demonstrated a >50% reduction in the resting gradient (13). In our study, the use of the CARTOSOUND system and ICE enabled more accurate delivery of RF energy. Nineteen of 20 patients underwent RF ablation successfully. The mean reduction of gradient is 47% after 6 months follow up, similar with prior reports. No serious complications, including vascular complications or permanent AVB occurred intra-procedurally or during follow up (9). In spite of the small number of case series identified from the literature, the preliminary results suggest RF ablation as an alternative treatment in some patients, especially in those who are unsuitable for ASA or surgical myectomy.

This study also demonstrated that the hemodynamic effects of the procedure are variable among patients. For example, a gradient reduction of only 3% was observed in patient No. 7 (Table 2 and Figure 5A). We further assessed factors that could influence the impact of this procedure in reduction of LVOT gradient (Figure 6). We found that the outcome of RF septal ablation is superior in patients with limited basal septal hypertrophy, shorter AML, and a normally positioned PM.

In patients with HOCM, increased LV wall thickness is most commonly located in the anterior free wall, basal septum and posterior portion of septum (20). The asymmetric septal hypertrophy narrows the LVOT, partially obstructing blood flow and the SAM of the mitral apparatus toward the hypertrophied septum lead to LVOT obstruction (3). Septal reduction therapy, such as surgical myectomy usually preferentially targets basal segment of the IVS to abolish Venturi forces, which are considered the major cause of SAM. For RF ablation, the lesion is mainly located in SAM-septal contact area, a similar target of myectomy. However, in some patients with diffuse septal hypertrophy, in addition to Venturi forces, overall disturbed blood flow also contributes to SAM (21, 22). The anterior-directed flow caused by mid-septal thickening can overlap with the posterior surfaces of the AML and push it to hypertrophied septum which results in obstruction. In this study, the patterns of interventricular sepal hypertrophy of 16 patients are recorded and analyzed. Besides the basal septum, the hypertrophied region can extend into mid or apical septum in whom the reduction of gradients is lower than others (Table 2 and Figures 4, 6), which emphasized the role of mid-septal hypertrophy. Ablation of the SAM-contact area is accurate and effective for patients with limited hypertrophy in basal septum but might be insufficient for patients with mid-septal hypertrophy. Extended myectomy has been designed and applied to redirect flow away from the mitral valve (23) and extended ablation targeting mid-septal segments need to be evaluated for patients in needs in the future. Szumowski (24) described a strategy of ablation from both sides of IVS to alleviate LVOTO gradient, which can be considered for further application in patients with extremely hypertrophied IVS.

In addition to left ventricular hypertrophy, structural abnormalities of the mitral valve and sub-mitral apparatus, such as leaflet elongation and anterior displacement of the papillary muscles also contribute to SAM pathophysiology (25). The average length of AML is longer in HOCM patients (34 vs. 24 mm). For those with an extraordinarily elongated AML (>30 mm), the residual portion often extends past the point of coaptation. Without the constraint of the LV–left atrium pressure difference, it can freely move with LV flow, even at low velocity. Late diastolic and early systolic flow then pushes the protruding leaflets into apposition with the septum (21, 22, 26). Meanwhile, the anterior displacement of PM exacerbates the magnitude of obstruction. There is closer proximity between the DPM and the hypertrophied septum especially the mid-septum and even contact each other at end of systole (Figure 2). This malformation positions mitral leaflets anteriorly into the flow stream cause a crucial overlap of the inflow and outflow portions of the LV that predisposes to SAM. Sherrid et al. indicated that a simple septal reduction procedure was not enough if AML > 30 mm and (or) DPM exist and resect—plicate—release (RPR) operation was required to alleviate LVOT gradients (25). Consistent with this observation, the reduction of gradient in patients with elongated AML or DPM is significantly lower (Figure 6). In our approach, RF energy was applied to create a localized reduction in contractility responsible for the dynamic obstruction. The maximal depth of penetration was measured by ICE and a mean depth of 4.3 ± 1.2 mm tissue damage was created during ablation. There was a non-significant reduction of the septal thickness after ablation. The structural abnormalities of mitral valve and papillary muscle are nearly impossible to rectify by RF ablation. Therefore, for patients with AMLs > 30 mm and (or) DPM, surgery with resect-plicate-release operation should be preferred.

For septal myectomy and ASA, the risk of permanent pacemaker implantation during follow up varied between 2.4–12.5 and 1.7–22.0%, respectively. In this study, the His and left bundle were annotated in the CARTO map before ablation in order to avoid injury to the conduction system and no permanent AVB occurred during follow up. After 6 months follow up, LBBB/RBBB persisted in two patients and the QRS duration was significantly prolonged in the overall cohort. In cases where the left branch bundle (LBB) courses directly through the SAM area, we intended to preserve the function of LBB which may affect the outcome of ablation. As for patient with prior RBBB, LBB preservation is more necessary. Patient No. 20 with a history of RBBB due to previous ASA developed into transient AVB several seconds after RF septal ablation. Methylprednisolone was administrated and AV conduction recovered soon after. Considering the high incidence of AVB, further ablation was aborted. In addition to the risk of AVB, tissue edema after RF ablation in SAM area may aggravate the LVOT obstruction transiently (14). Myocardial edema is well-recognized in CMR studies following RFA in other procedures (27). In this study, paradoxical increase of LVOT gradient after procedure was observed in eight patients. Methylprednisolone was administered for the following 3 days after ablation and edematous LVOT obstruction alleviated.

## Limitations

Firstly, this study was carried out in a single tertiary center and the follow-up period was relatively short. Secondly, limited by the small sample size, further analysis such as multiple linear regression was not performed. Thirdly, there was no comparison with surgery or ASA, but non-inferiority in effectiveness and safety was noticed with previous studies (13). Fourthly, there was lack of CMR data during follow up and no assessment of regional wall motion due to minimal septal ablation area. However, because of significant thickness of LV septum, RF lesion within SAM area was so shallow that might result in less hypokinesis there. Fifthly, we didn't pay attention to impedance drops during ablation.

## Conclusion

Endocardial radiofrequency septal ablation appears to be a safe and effective procedure for HOCM patients to alleviate the LVOT gradient, especially for patients with a shorter AML, limited basal septal hypertrophy and normally located PM.

## Data Availability Statement

The original contributions presented in the study are included in the article/supplementary material, further inquiries can be directed to the corresponding author/s.

## Ethics Statement

The studies involving human participants were reviewed and approved by the Medical Ethics Committee of Sir Run Run Shaw Hospital, Zhejiang University School of Medicine. The patients/participants provided their written informed consent to participate in this study. Written informed consent was obtained from the individual(s) for the publication of any potentially identifiable images or data included in this article.

## Author Contributions

CJ designed and supervised the study. QL, HQ, and CJ wrote the manuscript. All authors performed the study and analyzed data.

## Funding

CJ was supported by the Zhejiang Science and Technology Department: Operational Program: The establishment and clinical application of precise medical system in hypertrophic cardiomyopathy, registration no. 2019C03022.

## Conflict of Interest

The authors declare that the research was conducted in the absence of any commercial or financial relationships that could be construed as a potential conflict of interest.

## Publisher's Note

All claims expressed in this article are solely those of the authors and do not necessarily represent those of their affiliated organizations, or those of the publisher, the editors and the reviewers. Any product that may be evaluated in this article, or claim that may be made by its manufacturer, is not guaranteed or endorsed by the publisher.
